# Interprofessional training for final year healthcare students: a mixed methods evaluation of the impact on ward staff and students of a two-week placement and of factors affecting sustainability

**DOI:** 10.1186/s12909-015-0436-9

**Published:** 2015-10-26

**Authors:** Patricia McGettigan, Jean McKendree

**Affiliations:** 1 Barts and The London School of Medicine and Dentistry, Queen Mary University of London, Charterhouse Square, London, EC1M 6BQ UK; 2 Hull York Medical School, Heslington, York, YO10 5DD UK

**Keywords:** Interprofessional training, Interprofessional education, Evaluation, Barriers, Sustainability

## Abstract

**Background:**

Multiple care failings in hospitals have led to calls for increased interprofessional training in medical education to improve multi-disciplinary teamwork. Providing practical interprofessional training has many challenges and remains uncommon in medical schools in the UK. Unlike most previous research, this evaluation of an interprofessional training placement takes a multi-faceted approach focusing not only on the impact on students, but also on clinical staff delivering the training and on outcomes for patients.

**Methods:**

We used mixed methods to examine the impact of a two-week interprofessional training placement undertaken on a medical rehabilitation ward by three cohorts of final year medical, nursing and therapy students. We determined the effects on staff, ward functioning and participating students. Impact on staff was evaluated using the Questionnaire for Psychological and Social factors at work (QPSNordic) and focus groups. Ward functioning was inferred from standard measures of care including length of stay, complaints, and adverse events. Impact on students was evaluated using the Readiness for Interprofessional Learning Survey (RIPLS) among all students plus a placement survey among medical students.

**Results:**

Between 2007 and 2010, 362 medical students and 26 nursing and therapy students completed placements working alongside the ward staff to deliver patient care. Staff identified benefits including skills recognition and expertise sharing. Ward functioning was stable. Students showed significant improvements in the RIPLS measures of Teamwork, Professional Identity and Patient-Centred Care. Despite small numbers of students from other professions, medical students’ rated the placement highly. Increasing student numbers and budgetary constraints led to the cessation of the placement after three years.

**Conclusions:**

Interprofessional training placements can be delivered in a clinical setting without detriment to care and with benefits for all participants. While financial support is a necessity, it appears that having students from multiple professions is not critical for a valuable training experience; staff from different professions and students from a single profession can work successfully together. Difficulty in aligning the schedules of different student professions is commonly cited as a barrier to interprofessional training. Our experience challenges this and should encourage provision of authentic interprofessional training experience.

## Background

The complexities of healthcare require multiple skills to ensure best outcomes for patients [1, 2]. In medical training, educators are well aware that doctors must be both competent clinicians and effective members of multi-disciplinary teams (MDTs) that include other doctors, nurses, care assistants, therapists, and pharmacists. There is also a need to work effectively across specialty boundaries and between hospital and community settings. This view is embraced by healthcare policy makers and educators nationally and internationally and to optimise MDT-working, there have been calls for effective interprofessional training, both within the workplace among professionals and during pre-qualification [3, 4]. Unfortunately, multiple care failings in hospitals have highlighted poor team-working and prompted renewed focus on the necessity for effective interprofessional relationships and training aiming to improve the functioning of the MDT and a call for commissioning, governmental, professional and healthcare bodies to support interprofessional education development in the UK [2, 5, 6].

Interprofessional education that included hands-on clinical training began with the ‘Linkoping model’ in 1986 which incorporated a two week clinical working experience [7]. Other medical schools also established interprofessional training wards where pre-qualification students from different professions worked together alongside qualified staff, planning and providing patient care [8]. Well-developed in Sweden [9], these ‘working’ wards remain an uncommon feature of healthcare education despite encouragement [3]. Interprofessional education should incorporate practical experience, but concerns about inexperienced students delivering clinical care, patient safety, professional learning ‘cultures’ and especially, the costs and logistics of ensuring multiple healthcare professions are represented among participating students have impeded uptake [6, 10, 11]. Evaluations of interprofessional training in healthcare curricula have been generally positive but have focused on the views of patients and students rather than the experiences of ward staff involved with training [12, 13]. If practical interprofessional training is to be encouraged, then knowledge is needed about its impact on clinical staff and their capacity to maintain high quality care for patients while facilitating effective student training.

This study takes a novel, multi-faceted approach to evaluation of interprofessional training, investigating not only impact on students, but on staff and patients. The evaluation covers a timeframe from before the ward experience began through the delivery of the programme from 2007 and 2010 for three cohorts of undergraduate final year students at Hull York Medical School (HYMS) and for small numbers of final year therapy and nursing students.

## Methods

### Interprofessional training: placement duration, setting and supervision

The interprofessional training placements were of two weeks duration and were undertaken at Goole and District Hospital on Ward 2, a rehabilitation unit comprising eighteen in-patient beds, ten day-care places, and a falls clinic. Patients were generally 65 years of age or older, needing rehabilitation following events including stroke, fracture or surgery complications, and having multiple medical problems. Nursing care was provided by a stable cohort of nurses and care assistants. Many nurses were educational mentors, already practiced in supervising nursing students on training placements. Individually, they had specialist training in stroke care, tissue viability, diabetes management, infection control, moving and handling, nutrition, cardiology, and respiratory medicine. Medical care was supervised by two rehabilitation physicians and two staff doctors. Physiotherapy and Occupational Therapy were provided on site with a rehabilitation gym located on the ward. Nutrition and Speech and Language therapists attended twice-weekly.

All of the Ward 2 staff acted as facilitators for the students. Prior to commencing placements in August 2007, staff participated in preparatory workshops on interprofessional training, multidisciplinary team-working, the HYMS curriculum, and expectations of their roles as facilitators. Thereafter, new staff were inducted when they joined though this was uncommon because the staff base was stable. Two designated facilitators supervised placements, one HYMS-based (PMcG) and one ward-based, the Ward Sister during the first year, thereafter a senior nurse. Therapy teams each appointed an interprofessional training lead. Funding backfill was provided by HYMS for one nursing salary to compensate for ward-based facilitator time.

### Aim and objectives of the study

The aim of this study was to assess impacts on both clinical staff and students of delivering the interprofessional training placement in a working ward environment. Our objectives were to examine the effects on work demands and quality of care as perceived by staff, to assess standard measures of ward functioning, and to determine whether the experience influenced students’ attitudes toward interprofessional working in the MDT.

### Students

**Medical students**: The placement was a mandatory component of HYMS’ final year curriculum. It was undertaken in 2007–8 by 106 students, 116 in 2008–9, and 140 in 2009–2010. **Nursing and therapy students**: Support from nursing, occupational therapy and physiotherapy colleges led to inclusion of final year students in 2007–8 (*n* = 3 nursing students on 3-month placements), 2008–9 (*n* = 10 students; 6 nursing, 3 occupational therapy, 1 physiotherapy) and 2009–10 (*n* = 16; 8 nursing, 3 occupational therapy, 5 physiotherapy). Aligning complex schedules permitted only small numbers. Nursing and therapy placements in 2008–9 and 2009–10 were of 2–6 weeks duration and all students participated in inter-professional training activities alongside the medical students.

### Placement outcomes

There were four ‘generic’ learning outcomes, mapped to the curriculum outcomes for each profession: 1) Respect, understand and support the roles of other professionals involved in health and social care; 2) Demonstrate a set of knowledge, skills competencies and attitudes which are common to all professions and which underpin the delivery of quality patient/client –focussed services; 3) Deal with complexity and uncertainty; 4) Collaborate with other professionals in practice.

### Placement structure

Each placement included 8–12 students who worked in smaller groups of 3-4/shift on a 7-day-a-week roster with HYMS-funded accommodation provided on-site. Students were present on the ward for approximately fourteen hours/day during their two week placement, for a total of seven months/year; there was a one-week student-free ‘break’ every six weeks. Day 1 commenced with induction that included a discussion of interprofessional education and training, a patient moving and handling practical session, ward tour and staff introductions. Throughout the placement, each small group of students worked an early shift (07.20–14.45 h) or a late shift (13.30–21.30 h) or had a day off. On their shifts, students were teamed, usually in pairs with a nurse and a care assistant, each team being responsible for 4–5 patients. To gain understanding of other professions’ roles and skills, all students participated in all aspects of care, not just their own profession-specific activities. They negotiated with their colleagues as to ‘who did what’, for example, assisting with therapy sessions, going on home visits, undertaking tests and clinical observations, participating in medication rounds and ward rounds. All students contributed as needed in assisting patients with activities of daily living. Students and staff shared their skills, demonstrating to each other what their individual professional roles involved, and importantly, what they did not normally involve. At the same time, the primary business of the ward, patient care and rehabilitation, continued. A summary schedule is presented in Table 1 to help illustrate the activities undertaken during each shift.

**Table 1:** Outline of daily timetable for students working on the interprofessional training ward

Time	Early shift activities
07.20	Student sign in. Handover from the night shift.
07.35	Prepare patients for breakfast.
08.00	Give out breakfast, assist patients with feeding as required. Record food intake. Collect dishes. Bed making. Patient care; washing, dressing. Clinical observations. Morning medication round with registered nurse.
09.00	Tea/Coffee meeting to review overnight events, attended by multi-disciplinary team – handover; planning for the day. Occupational therapists and Physio decide priority order of patients needing therapy. Agree student attendance at therapy/home visits. Agree student medical tasks (eg blood tests, clinical examinations). Shared patient care and profession-specific clinical work; Liaise with staff doctors and nurse in charge for clinical queries. Documentation / patient notes to be completed. 15 min break to be taken during this time.
11.30	Escort able patients to dining room for lunch. Assist with feeding as necessary. Collect dishes. Record food intake.
12.00 – 13.00	Student lunch to be taken in 2 groups.
13.00 – 13.30	Prepare for handover to late shift team. Ensure registered nurses aware of any changes/developments in patients’ care to facilitate their taped handover to incoming staff.
Time	Late shift activities
13.30 – 14.00	Tutorial slot.
14.00 – 14.45	Handover early shift to late shift; led by students; facilitator and MDT members attend. Medication round at 14.00
14.45 - 15.20	Reflection period for early shift; facilitator attends.
14.45 - 16.30	Late shift students sign in. Provide shared patient care on the ward. Complete any outstanding tasks and/or clinical work from morning shift handover. Review any investigation results. Review of each patient from your professional perspective. Update patient clinical notes. Liaise with staff doctors regarding any outstanding medical issues. On formal Ward Round days, present & discuss your patients with the physician. Check & update draft patient discharge summaries. Prepare patients for tea – shared care activity.
17.00 – 20.00	Give out tea and assist patients with feeding. Collect dishes. Record food intake. Medication round 18.00 with registered nurse. Evening therapy to be undertaken with patients.
17.00 – 18.00	Student tea to be taken in 2 groups.
20.00 – 21.15	Patient family/visitor time for update/ discussion as needed of patient progress. Evening clinical observations – shared care. Review and update patient records and draft discharge summaries. Assist patients into bed. Medication round.
21.15	Night staff – 15 min handover. Student sign out.

A multidisciplinary team meeting was held at 9 am daily to plan patient care. By the end of Week 1, early shift students were able to lead handover of their patients to the incoming late shift. Daily tutorials took advantage of skills among both students and ward staff; topics included pressure area care, nutrition, feeding, infection control, prescribing, fluid management and discharge planning. On the final day, the entire student group and facilitators met to reflect together on the experience, consider how the learning outcomes had been met, and evaluate benefits and drawbacks of the experience. Students completed the RIPLS questionnaire for the second time along with an evaluation of the ward experience.

### Governance and ethics

The placement was approved by HYMS, the hospital Board of Governors and the local NHS Trust. All patients (or their guardians) consented for student care. They were provided with written as well as verbal information and were made aware that consent could be withdrawn.

To ensure awareness about the students among visitors to Ward 2, posters describing the ward’s training role were displayed prominently at the entrance and in all common areas. Ethics approval to use evaluation data was granted by HYMS Ethics Committee and all students and staff were informed that aggregated data would be used for evaluation and research purposes.

#### Staff evaluations

Two activities were undertaken to assess the impact on staff of undertaking the interprofessional training role.

1. The Questionnaire for Psychological and Social factors at Work (QPSNordic) [14]: This 123-item validated questionnaire measures psychological and social factors at work, including job and organisation characteristics and individual work-related attitudes. Seven of its 26 subscales (relating to work-place demands, role clarity, and support) were relevant to our study and are reported here. Items were answered on a1 to 5 scale where 1 indicates disagreement (e.g. never/seldom) and 5, agreement (e.g. always/very much). It could be completed anonymously if desired and was distributed to staff in May 2007 and December 2007 in order to assess the effects on staff before and then shortly after the placements began.
2. Staff focus groups: Three focus groups were convened representing staff groups with whom the students worked on a daily basis. The first met before the students arrived (May 2007), the second on completion of the first year of placements (April 2008). In the first focus group, participants were asked to discuss their expectations, fears and anticipated benefits and disadvantages in having students working on Ward 2. In the second and third, participants were asked to reflect on their experiences, how these had or had not matched their expectations, and to describe the benefits and disadvantages for ward functioning, for patient care and for themselves as professionals.
  1. Each focus group lasted around one hour, led by two trained facilitators who were independent of the placement, and each focus group included five staff participants chosen to represent a range of professions. Nursing staff, care assistants and therapists each nominated at least one participant. All groups were audio recorded and were transcribed verbatim for analysis. We were interested in extracting common themes from the group discussions rather than concentrating on individual experiences, and in comparing the experience before and after the students arrived, so we developed a framework analysis to focus on the main questions of interest to our evaluation [15]. These orienting goals of the analysis were to highlight *concerns and expectations* before the students arrived and then to explore the *lived experience* and any *unexpected effects* after the placements commenced.

#### Ward evaluations

To evaluate the impact on clinical care, standard measures gathered by the hospital Trust were examined including records on adverse/critical events, medication errors, discharge-letter completions and complaints before and after annual student placements. We did not formally survey patients or families.

#### Student evaluations

Two activities undertaken to evaluate the student experience are reported in this study.

The first was the Readiness for Interprofessional Learning Survey (RIPLS),completed on Day 1 prior to induction and at the end of the placement [16]. Pre- and post-placement questionnaires were matched and score differences analysed. RIPLS is a validated questionnaire consisting of 23 statements each rated on a 5-point Likert scale (1 =strongly disagree to 5 =strongly agree) and scored in three domains: teamwork and collaboration, professional identity and patient centredness.

1. *Teamwork and collaboration* measures the link between the positive outcomes of team-working and adopting a team-based approach with effective communication and a willingness to share knowledge and skills (13 statements, minimum score 13, maximum score 65). A high score indicates a more positive attitude.

*Example statement*: I would welcome opportunities to work in interprofessional small-group projects

2. *Professional identity* acknowledges the importance of professional identify and an awareness of conflict between professions along with a readiness for inter-professional learning (5 statements, minimum score 5, maximum score 25). A low score indicates lessening of ‘professional silos’ and the realisation of contribution from all professions to good patient care.

*Example statement*: Clinical problem-solving should only be learned within my own profession / discipline

3. *Patient centredness* indicates an orientation to the patient’s needs rather than their own (5 statements, minimum score 5, maximum score 25). A high score indicates increased patient centredness.

*Example statement:* In my profession, one needs skills in interacting and co-operating with patients

The second activity was a questionnaire evaluation of the placement completed anonymously by students at the end of the placement. Intended to inform curriculum development, it was created at HYMS and comprised statements rated on a Likert scale, 1 (=strongly disagree) to 5 (=strongly agree) and a Yes/No question asked students if they thought the placement was a valuable experience. Table 2 shows the questionnaire items.

**Table 2:** RIPLS scores of medical students and of nursing and therapy students: pre- and post-placement scores and score changes

RIPL Scale	Pre-placement score	Post-placement score	Change score	T-value
	Mean (SD)	Mean (SD)	Mean (SD)	(*P*-value)
TEAMWORK (Max 65)				
Medical Students				
2007-08 (*n* = 99)	54.4 (8.1)	58.1 (6.9)	3.7 (6.6)	−3.42 (<0.001)
2008-09 (*n* = 86)	52.2 (6.7)	56.1 (7.1)	3.9 (5.8)	−8.66 (<0.0001)
2009-10 (*n* = 124)	53.2 (6.0)	56.5 (6.2)	3.3 (6.2)	−5.6 (<0.0001)
Nursing & Therapy Students				
2008-9 (*n* = 10)	59.6 (4.1)	62.9 (1.6)	3.3 (3.1)	−3.4 (<0.01)
2009-10 (*n* = 14)	56.1 (5.7)	60.2 (4.3)	4.6 (4.4)	−3.95 (<0.01)
PROFESSIONAL IDENTITY (Max 25)				
Medical Students				
2007-08	10.4 (2.8)	9.1 (3.1)	−1.3 (2.7)	4.0 (<0.0001)
2008-09	10.3 (2.8)	9.4 (3.2)	-.95 (2.8)	3.16 (<0.0001)
2009-10	10.8 (2.6)	10.3 (3.1)	-.41 (3.3)	1.32 (0.189)
Nursing & Therapy Students				
2008-9	9.6 (1.5)	6.4 (1.2)	−3.2 (0)	5.4 (0.0004)
2009-10	10.4 (2.5)	8.3 (1.9)	−2.1 (−2.6)	3.43 (0.004)
PATIENT CENTREDNESS (Max 25)				
Medical Students				
2007-08	23.1 (3.1)	23.5 (3.2)	.44 (3.3)	−1.06 (0.29)
2008-09	22.7 (2.2)	23.6 (2.0)	.84 (2.2)	−3.9 (<0.0001)
2009-10	22.9 (2.0)	23.6 (1.8)	.69 (2.2)	−3.29 (<0.001)
Nursing & Therapy Students				
2008-9	23.5 (2.3)	24 (2.0)	0.5 (1.6)	−1 (0.34)
2009-10	22.7 (2.1)	23.5 (2.0)	0.8 (1.6)	−1.86 (0.08)

Note: For teamwork and patient centredness, higher scores indicate better readiness; for professional identity, lower scores indicate better readiness

## Results

Between 2007 and 2010, placements were undertaken by 362 medical students and 26 nursing and therapy students.

### Impact on staff

#### Questionnaire for Psychological and Social factors at work (QPSNordic)

The QPSNordic questionnaire was completed by 33 staff members (100 %) during the initial training workshops in May 2007, before the placements had begun, and by 16 (48 %) in December 2007 after two months of placements. This permitted assessment of disruption to ward staff after a short period, but long enough to have ironed out any initial issues with organization. Table 3 summarises results from the seven subscales relevant to placement activities.

**Table 3:** Scores for ward staff on subscales of the QPSNordic Questionnaire for Psychological and Social factors at work

Subscale	Survey Date	Number	Mean score out of 5
Quantitative Demands	May 07	27	3.06
(workload, too much to do)	Dec 07	14	2.81
Decision Demands	May 07	32	3.77
(quick or complex decisions)	Dec 07	16	3.77
Learning Demands	May 07	33	2.67
(too difficult, need new skills)	Dec 07	16	2.60
Role Clarity	May 07	33	4.55
(clear objectives, responsibilities)	Dec 07	15	4.49
Positive Challenge	May 07	32	4.40
(challenging work, meaningful)	Dec 07	16	4.40
Support Manager	May 07	33	4.23
(support from manager, appreciated)	Dec 07	16	4.38
Support Colleagues	May 07	33	4.20
(support from co-workers)	Dec 07	16	4.21

Note: All differences in scores were non-significant. Some questions were not answered by all respondents, so the numbers (N) contributing to individual subscales vary slightly. (1 = strongly disagree, 3 = neither agree nor disagree, 5 = strongly agree)

An ANOVA examining all sub-scales at both time-points indicated no significant differences between the May (before students) and December (after students arrived) responses. Neither the overall ANOVA nor individual t-tests for the subscales showed significant before/after differences.

#### Focus groups

The framework analysis of the focus groups highlighted *concerns and expectations* before the students arrived and explored the *lived experience* and any *unexpected effects* after the placements commenced.

1. Pre-placements focus group: concerns and expectations

Three themes emerged from the participants before the students arrived around the *concerns and expectations* of the staff on the ward: *enthusiasm, apprehension, trust*:

1. Enthusiasm: the students presented an opportunity to learn and to improve knowledge and patient care.
2. Apprehension: Were the staff ‘up’ to the task? Would it disrupt good patient care? Would it disrupt good working relationships? Would some staff roles be displaced by the students (particularly care assistants)?
3. Trust: The Ward manager committed them because she knew the team was strong already and would meet the challenges

2. Post-placements focus groups: lived experience and unexpected effects

Again, three main themes emerged around perceptions of *the experience* and *unexpected effects*: *enjoyment, learning, pride in the level of patient care.* These were similar across both April 2008 and 2010 groups.

1. Enjoyment: Placements were more fun than expected despite the onus of supervision. Final year students were quite skilled clinically but insightful of their own limitations and were able to recognise when they needed assistance or instruction from ward staff colleagues which quickly allayed early concerns about maintaining a high quality of care.
2. Learning in both directions: Though placements were hard work because students were present on the ward from 7.30 am until 9.30 pm seven days a week for practically seven months each year, there was a two-way learning flow. By participating in nursing and therapy activities, staff felt that medical students in particular improved their own profession-specific skills. They shared their skills in turn, explaining for example why particular tests were needed and demonstrating or explaining clinical signs. While ward staff gained from this, these exchanges were deemed especially rewarding when students from several professions were working together.
3. Pride in Ward performance: Patient care benefited because students actively contributed to care-giving. They asked many care-based questions, so staff continually considered the rationale for what they did. Care assistants’ concerns of displacement were not realised; in working alongside students they had more time with patients and felt this improved the care they provided. Students were instructed to spend time with patients and family at visiting times and it turned out that this unrushed access to members of the caring team was greatly valued by families.

The consensus from both focus groups was that interprofessional training was a positive experience for staff. In particular, it made staff conscious of public perception or ‘how others see us’, in the words of one participant and this was felt to be a benefit, encouraging consistently high standards of care.

#### Ward evaluations

Of around 500 patients cared-for during student placements, one refused consent for student care. There were no withdrawals of consent or complaints about the students. There was one significant medication-related adverse event which was reported as a critical incident. A drug was administered twice with neither student nor nurse doing the medication round noticing it had already been given. It did not cause harm to the patient who was told of the error.

The length of stay was stable throughout the placement periods. Students were responsible for drafting discharge letters that were subsequently screened and completed by the staff doctors. Rates of completion improved from 50–60 % completed on the day of discharge when the students were not on placements to 95 % during placements.

#### Impact on students

RIPLS scores are reported in Table 2. For medical students, the numbers of matched pre- and post-placement scores were 99 of 106 (2007–8 cohort), 86 of 116 (2008–9), 124 of 140 (2009–10), in total, 308/346 (85 %) matched scores; for nursing and therapy students 10 of 10 (2008–9) and 14 of 16 (2009–10). (The three nursing students undertaking 3-month placements during 2007–8 worked alongside multiple groups of medical students. Owing to the extreme difference in placement duration, we did not ask them to complete the RIPLS.)

Table 2 presents the results for each cohort of the medical and the nursing and therapy students.

Scores on the Teamwork scales increased following the placement for both medical student and nursing and therapy student groups. Scores on Professional Identity decreased significantly for all but one medical student cohort. Changes in Patient Centeredness were small though pre-test scores were already high among all cohorts.

The responses for all three medical student cohorts on the medical student placement evaluation questionnaire were very similar and were therefore combined (Table 4). Students considered that two weeks was adequate to gain understanding of interprofessional working. Overall, they rated the placement highly. Most considered the experience valuable.

**Table 4:** Medical student placement evaluation survey: statements and rating scores

End of placement medical student evaluation	Rating score 1–5
	*N* = 362
I enjoyed the Training Ward experience.	4.03
Working in teams has helped me to achieve the learning outcomes.	4.17
Ward Facilitators have helped me to achieve the learning outcomes.	4.15
The permanent staff on the ward helped me to achieve the learning outcomes.	4.20
Continuous access to 'real' patients has helped me to achieve the learning outcomes.	4.26
The staff that I encountered on the placement were helpful and supported the learning process	4.33
The experience of the Training Ward has informed my understanding of interprofessional working.	4.41
The experience of the Training Ward will have a positive effect on how I work with healthcare colleagues in the future	4.2
The duration of the placement, 2 weeks, is adequate to gain an understanding of interprofessional working	4.3
A 3-week placement would have been of greater benefit in gaining an understanding of interprofessional working	2.0
Provision of on-site accommodation was important for managing attendance at rostered shifts	4.53
Overall, do you feel that the Training Ward placement has been a valuable experience? (Y/N)	339 Yes (94 %)
	23 No (6 %)

Note on scoring: 1= strongly disagree, 3= neither agree nor disagree, 5= strongly agree

## Discussion

To our knowledge, this is the first report on interprofessional training that takes a strong multi-perspective view on evaluation, planned before the ward started, to consider the effects on the staff delivering the training and the ward outcomes as well as on the students undertaking it. Our evaluations demonstrate that staff delivering their roles as interprofessional training facilitators had a very positive experience in terms of their perceptions of professional pride, skills recognition, care quality, and demonstration of their capacity to share expertise with the students. Both before and after students arrived on the ward, QPSNordic evaluations showed that there was sometimes a high workload, staff felt well supported by managers, their work roles and responsibilities were clear and challenges were viewed as positive and meaningful. There were no significant shifts in QPSNordic scores indicating that there was not a significant negative impact on workload or morale of having the students, concerns voiced in the pre-placement focus group.

Student supervision did not divert time from patient care. Staff reported more time to spend with patients because students actively contributed to care. Though students needed continual supervision, this did not cause the disruption feared in the first focus group. This was supported by the absence of complaints about the students, a stable length of patient stay, improved discharge letter completion rates, and almost universal consent for student participation in care with no withdrawals of consent. There was one serious medication incident that did not result in patient harm.

The RIPLS results showed increases in students’ understanding of the roles of other professionals, willingness to adopt a team-based approach to sharing knowledge and skills within the multidisciplinary team, and a lessening of the “professional silo” attitudes known to be destructive to good team working [17]. Post-placement team-work scores improved significantly compared with pre-placement scores. All cohorts had similar pre-placement professional identity scores and improvement was shown by score reductions for all but one cohort. Owing to small numbers of nursing and therapy students, caution is needed in comparing their results with those of the much larger medical student cohorts. Their scores however suggest they entered better prepared for teamwork and with greater professional identity awareness, but still improved significantly on both counts. This suggests some aspects of their prior training had better-prepared them for interprofessional working, but the authentic experience still offered benefit. This may reflect greater integration (compared with medical students) in the uni-professional teams during prior placements where they are ‘workers’ embedded in their teams rather than ‘observers’ like medical students. All students showed high pre-placement levels of patient-centeredness that were maintained.

Medical students’ evaluations of the placement, undertaken to inform curriculum development, were highly supportive. This surprised us because the two-week placement represented time taken ‘out’ of a 6-week medicine, surgery, or general practice placement and we knew some students anticipated this would have a negative impact on that learning. Nevertheless, 94 % rated it a valuable experience.

Previous studies of ward-based interprofessional training have found a positive impact on students’ attitudes and views and our findings are in agreement [9, 18]. However, positive impact is not guaranteed [19]. Many factors influenced the success of this interprofessional experience including structure, particularly the opportunity both to observe and participate in authentic team-based clinical settings rather than only learning together in a classroom, facilitator enthusiasm and commitment, shared status and vision, and institutional funding and support [20, 21].

While these conditions were achieved on the training ward during this period, there were challenges. The placement ceased in 2010 owing to capacity issues. Increasing numbers of medical students made it impossible to offer a 2-week placement to everyone within the time constraints of the academic year. Medical student numbers per placement increased from 8/group in 2007–8 to 10-12/group in 2009–10 giving an excessive ratio of students to staff on some shifts, especially noticeable on the few occasions when the ward was not full to capacity. Maintaining smaller groups would have required securing a second placement ward with associated increased running costs. This was deemed unsustainable and the decision was taken by HYMS to end the placement for final year students. An interprofessional training placement was subsequently offered as a self-selected component of Year 3 work so the experience remained available for small numbers of more junior students.

On a practical level, while funding problems ended HYMS’ interprofessional training placement, the absence of students from other professions was not a critical issue though their presence was desirable and we invested considerable work in ensuring as many as possible undertook placements. Timetabling logistics were the main impediment and during three years, there were 362 medical students and just 26 nursing and therapy students. The small numbers meant that medical students were working interprofessionally with the ward staff for the bulk of their time. Not only did the students benefit, the experience was valuable for staff too, challenging assumptions that to be successful and sustainable, interprofessional training necessarily requires *students* from multiple professions to work together.

## Limitations

Undertaking an evaluation as complex as this in a clinical setting has inevitable limitations. Despite nursing and therapy collaborator support, the numbers of students from professions other than medicine were small owing to the scheduling complexitiesso we could not gain a reliable picture of the effects that students from multiple professions had on each other. Nevertheless we found that medical students working mainly alongside trained staff from multiple professions consistently showed significant gains in RIPLS domains thereby supporting a true benefit from the experience. Staff completion of the second QPSNordic survey was just 48 % so it is possible negative views were missed. However, the survey was completed anonymously so we feel this is unlikely. Furthermore the focus group findings support a generally highly positive staff view of the placement. Our study did not assess if the placement impacted on real-life interprofessional working after the medical students graduated. One impediment was the absence of an appropriate instrument to assess doctors’ inter-professional team-working skills. We developed and validated a suitable instrument [22] but owing to small numbers of HYMS graduates assigned to local hospitals as Foundation Year 1 trainees, reliable determination of the placement impact on actual practice was not possible.

## Conclusions

We have shown that in delivering interprofessional training, it is possible to integrate large numbers of students alongside clinical staff to provide hands-on patient care in a working ward environment without detriment to standards of care and with benefits for staff and students. Our experience demonstrates that a common impediment, the difficulty of ensuring representation of students from multiple professions, can be overcome, though a commitment to properly funding and organising wider health professional education to encourage interprofessional learning and working continues to be a desirable goal.
